# Experimental Study on the Interlaminar Fracture Properties of Carbon Fibre Reinforced Polymer Composites with a Single Embedded Toughened Film

**DOI:** 10.3390/polym13234103

**Published:** 2021-11-25

**Authors:** Evanthia J. Pappa, James A. Quinn, James J. Murray, James R. Davidson, Conchúr M. Ó Brádaigh, Edward D. McCarthy

**Affiliations:** School of Engineering, Institute for Materials and Processes, Sanderson Building, The University of Edinburgh, Robert Stevenson Road, Scotland EH9 3FB, UK; j.quinn@ed.ac.uk (J.A.Q.); j.j.murray@ed.ac.uk (J.J.M.); j.r.davidson@ed.ac.uk (J.R.D.); c.obradaigh@ed.ac.uk (C.M.Ó.B.); ed.mccarthy@ed.ac.uk (E.D.M.)

**Keywords:** mode I fracture, mode II fracture, films, interlaminar toughness, secondary bonding, co-curing, carbon fibre

## Abstract

In this study, two types of single polymer films have been inserted in a composite laminate to examine their toughening effects on mechanical properties. The first is a thermoplastic polyurethane (PU) film, and the second is an adhesive epoxy film featuring a polyester net. The laminates were manufactured either using a co-curing (CC) process or a secondary bonding (SB) process used for the epoxy film. Mode I and mode II interlaminar fracture toughness were measured for laminates manufactured by both processes and compared with the corresponding reference laminate toughness. A significant increase in both mode I and mode II toughness resulted when introducing a single PU film, approximately 290% and 50%, respectively. Similarly, the epoxy film improved the interlaminar fracture properties; the CC process produced an increase of 175% for mode II toughness, while the SB adhesive film showed an increase of 75% for mode II toughness.

## 1. Introduction

Composite materials have been increasingly used in primary load-bearing structural applications over the last two decades. Unlike metals, the microstructure of a composite gives rise to complex modes of failure that can be difficult to characterize [1,2]. Carbon fibre reinforced polymer (CFRP) composites have been established as engineering materials in many critical failure applications such as aerospace, transport and marine applications. In such applications, the laminated composites can experience impact damage, which can cause an ultimate failure process [2,3,4,5]. Composite designs withstand in-plane loads effectively, so that most damage in composite structures is associated with impacts and out-of-plane stresses [4,5,6,7]. Failure usually occurs progressively through a variety of damage mechanisms, without initially causing catastrophic failure of the structure. There are three different kinds of crack failure which can occur in laminated composites: interlaminar, intralaminar, and translaminar failure [1,8]. The interface between the fibre and matrix affects the performance of the composite and plays a significant role in critical forms of damage such as delamination, which reduces the load-carrying capability of the structure [9]. Delamination is defined as a crack that occurs between two plies in a composite structure. The resistance of a composite structure to delamination cracking can be characterized using the fracture toughness or the fracture strength of the material. Fracture toughness is dominated by the matrix of the composite laminate; therefore, it is important to obtain good adhesion properties to achieve high fracture toughness [2,5,6,7,8]. In the literature, there are many studies on adhesive joining and toughening of CFRP laminates, which address the need for improved bonding properties, joining strategies and toughening of composites for high-energy impact absorption.

Toughening a composite material is a common tactic for enhancing damage resistance. There has been much interest in inserting thermoplastic veils to improve interlaminar fracture toughness [10,11,12,13], especially since the commercialization of Xantulayr^®^, a toughening veil made of electrospun nanofibres by Revolution Fibers [14]. Depending on the type of fibres, soluble or insoluble, thermoplastic veils can result in agglomerates when the dispersion is poor. A soluble veil can be a nanoparticle carrier and result in agglomerates that weaken the composite structure, unlike an insoluble veil [15]. Several studies have developed custom interleaves that significantly increase CFRP interlaminar fracture toughness [16,17,18,19,20]. Arai et al. [21] embedded nanomodified films in CFRP and reported about 200% higher mode I fracture toughness than for a pure CFRP laminate. Others have also reported higher mode I and mode II interlaminar fracture toughness by using interleaves of different polymeric structures [22,23,24,25,26,27]. The development of custom interleaves with intricate heterogeneous structure (veils or sophisticated nanoparticle systems) using higher cost precision fabrication techniques is gaining attention, however, the object of the present study is to investigate whether the same toughening effects can be achieved with existing homogeneous, less expensive off-the-shelf commercial single ply films. Polyurethane (PU) is a polymer that is commonly used as a foam or matrix. Only a few studies found in the literature use PU films or nets for energy absorption properties [28,29,30,31,32]. Kishi et al. [31] used several types of films, including polyurethane, in multiple locations in a laminate to enhance the damping loss factor. The latter increased by a factor of two at multiple frequencies where PU films were used, but at the expense of lower flexural properties compared to the control CFRP, i.e., a decrease in modulus from 120 GPa for the neat CFRP control to 21 GPa for the PU-laminate. In a later study, Rizzo et al. [32] used polyurethane films as coatings on the top of CFRP laminates to improve their impact resistance, resulting in lower internal damage for railway applications. Although polyurethane films have been used with laminates previously (as alternating interleaves or surface films), there is, to the best of our knowledge, no published description of a laminate with a single embedded PU film, which introduced in this paper.

When studying the increase in interlaminar fracture toughness achieved by adhesive films, there are three fabrication processes reported in the literature: co-curing (CC)—all parts are co-cured together under the same curing conditions; co-bonding (CB)—at least one of the parts is cured when bonding with other uncured parts; and secondary bonding (SB)—the only uncured part is the adhesive. Mohan et al. [33], used an FM300-2M adhesive film to compare adhesion and mode I toughness properties obtained from laminates manufactured in a co-curing process and a secondary bonding process. The study showed a high dependence on the type of starter defect and the type of joint; meaning co-cured versus secondary bonding. A similar adhesive was studied by Balzani et al. [34], who examined the toughness of high-performance unidirectional (UD) laminates using both the double cantilever beam test and end-notch flexure test where a cohesive failure was observed uniformly distributed over the interfacial fracture surface. They also reported some areas where the crack path switched into the composite. Tavara et al. [35] examined the interfacial crack propagation of different adhesives and the effect of polyester in promoting non-smooth behaviour of mode I fracture. Other studies on failure mechanisms in adhesive films focused on adhesives where cohesive failure was obtained due to peeling and/or shearing forces [36,37,38,39,40,41]. Although inserting films can have very positive effects on adhesion, Jiang et al. [42] reported a negative effect with decreased values of mode I of more than 70% and poor adhesion when embedding polyethylene terephthalate (PET) film in a UD CFRP laminate. A key question when manufacturing CFRP with an adhesive film is whether co-curing the CFRP and the film integrally during cure is a more effective process than secondary bonding of the film between two pre-cured laminates. In the current study laminates using a single epoxy adhesive film, featuring a polyester net, were fabricated using both CC and SB methods, and the comparative toughness results are presented.

Due to the need for large-scale applications, good bonding and damage resistance are essential characteristics of high performance CFRPs. The currently increasing volume demand for composite materials and the cost and rate limitations of the autoclave process, alternative out-of-autoclave (OoA) manufacturing techniques, such as the compression moulding are being considered for medium to high volume production where high initial tooling cost can be offset [43]. Where such cost cannot be borne, however, vacuum-bag only processes are used, but due to the limited consolidation pressure available (1 bar), relatively high void percentage, and porosity, extensive delaminations and poor ply adhesion can result [7,44,45]. In the current study, OoA laminated composites have been manufactured in a compression moulding process to obtain high quality laminates and to demonstrate that both the examined films could be effectively used in a high-volume process.

The purpose of this study is to examine the interlaminar properties and the toughness of CFRP laminated composites with single embedded films compared with the neat CFRP laminates. For this purpose, two films were examined: (1) an aliphatic polyurethane film used in a CC process; and (2) a toughened epoxy adhesive film, inserted via both CC and SB processes; to compare the difference in toughness between these film bonding techniques. Mode I and mode II interlaminar fracture properties have been measured using the double cantilever beam (DCB) and end-notch flexure (ENF) tests. The specimens’ delamination morphology and fracture surfaces were observed using scanning electron microscopy (SEM) and a high-resolution camera.

## 2. Materials and Methods

### 2.1. Processing and Preparation

#### 2.1.1. Materials and Manufacturing Processes

**Figure 1:**
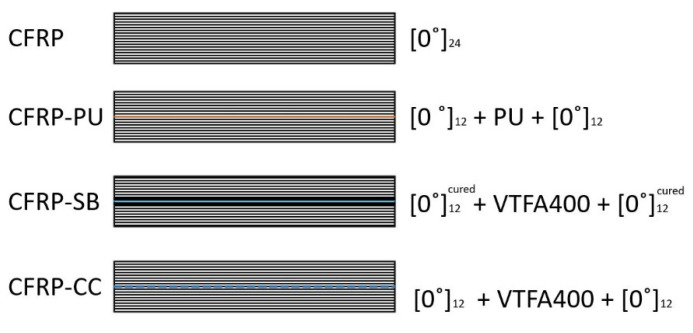
Lay-up illustration of the different examined cases.

Composite laminates were produced in a LAB 450 PEI Pinette heated platen press. The temperature was controlled and recorded throughout the curing process. Pre-impregnated (pre-preg) T700 carbon fibres (Toray) combined with a VTC401 epoxy matrix, supplied by SHD Advanced Composite Materials Ltd., UK, were used for the fabrication of the panels. A 25 μm thick thermoplastic polyurethane film, supplied by Schweitzer-Mauduit International (SWM) Ltd., UK, was co-cured within the pre-preg to produce CFRP panels with the film, CFRP-PU. A 0.2 mm thick VTFA400 adhesive film, supplied by SHD Advanced Composite Materials Ltd., UK, was used to produce co-cured laminates, CFRP-CC, and secondary bonded laminates, CFRP-SB. The VTFA400 is an epoxy with a polyester net to maintain the thickness. In all cases, a standard manual lay-up process was followed to produce bulk panels of 300 × 300 mm planar dimensions, Figure 1.

A total of 24 plies was used for each of the manufactured laminates, in order to achieve a thickness between 4–5 mm. For better consolidation of the panels, a stage-wise de-bulking process was implemented. All CC plates, CFRP, CFRP-PU and CFRP-CC were cured using the same temperature ramp rate of 3 °C/min rate with an applied pressure of 5 bars. The maximum curing temperature was 120 °C, where the laminates were cured for 45 min. For the CFRP-SB, two CFRP laminates (12 plies each) were first cured and then joined together with the VTFA400 epoxy adhesive film in a post-cure process, at 120 °C, with a much lower 0.3 °C/min heating rate and a pressure of 1 bar; following the manufacturer’s guidelines for post-curing. Compression moulding was used for the manufacture of the laminates. Following the supplier’s instructions for the pre-pregs, the curing process of the CFRP results in a glass transition temperature of ca. 118 °C, established by dynamic mechanical analysis (DMA) measurements.

#### 2.1.2. Sample Preparation

Strips of 2 mm from all sides were cut from the cured laminates to avoid edge defects. Rectangular specimens were cut with a water-cooled diamond tip saw, following the standard for each test case. The specimens were cut parallel to the direction of fibres. Due to the water used during machining, an environmental chamber was used to dry the specimens at 50 °C for 16 h prior to the test.

#### 2.1.3. Reliability, Accuracy and Validity

Experimental results are strongly dependent on manufacturing quality. Thus, to ascertain laminate quality after the curing process, all the laminates were tested with an ultrasound scanner, DolphiCam supplied by JR Technology Ltd., UK, to avoid testing laminates with voids (above 3%), delaminations, or other imperfections. The thickness of each plate was measured with a gauge meter. From different locations of each laminate, 3–4 rectangular specimens of 5.00 mm × 7.00 mm were machined to measure the density and fibre volume fraction. A burn-off process (ASTM D2734) was performed to calculate the fibre volume fraction (FVF). Table 1 presents the thickness, the density and the FVF, for all the cases: CFRP, CFRP-PU, CFRP-SB and CFRP-CC.

### 2.2. Testing

The experimental study of the manufactured laminates focused on the interfacial properties and the effect of the films on the fracture toughness of the composite. For every laminate case, a total number of 6 test coupons each were used to measure mode I and mode II fracture toughness. In all cases, a single film was introduced as the middle ply of the composite. The crack insert used for both DCB and ENF tests was a Teflon PFA film supplied by Lohmann Technologies (UK) with a thickness of 12.7 µm. The Teflon PFA film was inserted during the manufacturing process for all the examined laminates. According to both standards, the width of the specimen was 25 mm and the thickness was between 4.00 mm and 5.00 mm. All the tests were performed using an Instron 3360 test frame. For the DCB and the ENF experimental tests, the delamination growth was determined in real time, using a video gauge extensometer supplied by Imetrium Ltd., UK. Finally, high resolution photographs and scanning electron microscopy (SEM) images were taken to investigate the fractured surfaces, to illustrate the differences between the fractured laminates as a function of the manufacturing and bonding process.

#### 2.2.1. Double Cantilever Beam Test (DCB)

The interlaminar mode I fracture toughness was measured for all the cases using a DCB experimental set up. The test was carried out following the ASTM D5528 standard with a 10 kN load cell and a crosshead displacement of 1 mm/min. The compliance of the machine was considered according to the standard. For the execution of the test, two cubic metallic blocks were used for each specimen, which were bonded on either side of the pre-cracked end of the specimens. The adhesive used to bond the blocks with the specimens was a MTFA400 adhesive film supplied by SHD Advance Composite Materials Ltd., UK. The crack initiation length for all the specimens was in the range 64–70 mm. Digital image correlation (DIC) marks were noted over the length of the specimens to record the crack propagation of each specimen using the video gauge extensometer. The final crack extension point was ca. 50–60 mm distant from the crack initiation point towards the other end of the specimen.

#### 2.2.2. End Notch Flexure Test (ENF)

The interlaminar mode II fracture toughness was measured for all the cases using a three-point end notch flexure test. The tests were carried out following the ASTM D7905 standard, with a 10 kN load cell. The dimensions of the spans followed the test standard for a three-point bending test fixture. For all the specimens, the crack initiation was between 60 and 65 mm. For the compliance calibration, the loading rate was 0.8 mm/min while the unloading rate was 1.6 mm/min. For the estimation of the mode II fracture toughness, two separate calibration loading cases were used: in the beginning, the non-pre-cracked (NPC) calibration curve, (gives initial crack toughness), and then the pre-cracked (PC) calibration curve, (gives secondary crack toughness). In the NPC approach, the insert was used to create a pre-crack that was the initial point for the PC, within the minimum toughness plateau. The crack point on the specimen was estimated and observed by a stereo microscope, Stemi 2000-C, supplied by Carl Zeiss AG, Germany. A video gauge extensometer was used for the recording of the crack propagation throughout the experimental procedure using the DIC marks. The final crack propagation was in the range of 30–40 mm from the secondary tip propagating point towards the other end of the specimen.

#### 2.2.3. Scanning Electron Microscopy (SEM)

Fracture surfaces for each material and test configuration were imaged using a Hitachi TM4000 Plus tabletop scanning electron microscope (SEM). Rectangular samples were cut from tested specimens by extracting an area between 15 mm and 25 mm from the initial pre-crack location, e.g., the stable crack growth region. Specimens were then dried in an oven at 50 °C for 1 h. Mixed images of both secondary electrons and back-scattered electron signals were produced at a 5 kV accelerating voltage. Since a magnification of only 2000× was required, surface-charge build-up prevention techniques such as sputtering or carbon coating were not needed [46].

## 3. Results

### 3.1. DCB Results

**Figure 2:**
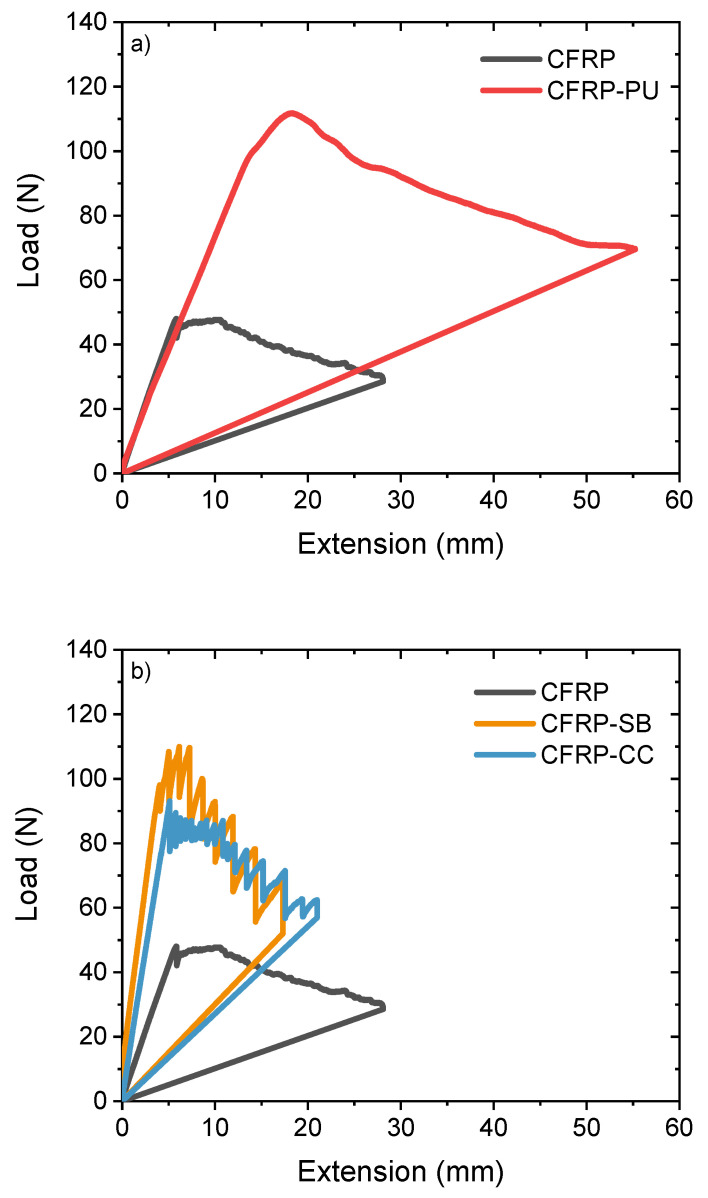
DCB R-curves (**a**) CFRP compared with the CFRP-PU and (**b**) CFRP compared with both the CFRP-SB and CFRP-CC.

The R-curves for the DCB tests were obtained from the experimental force-displacement, P-δ, curves, considering the data reduction method proposed in the test standard. The introduction of the films has significantly increased the values of mode I interlaminar fracture toughness. From the results obtained, an increase in mode I fracture toughness of ca. 290% was observed for the CFRP-PU specimens compared with the reference CFRP specimens; however, there was no significant increase in the mode I toughness for the CFRP-CC and CFRP-SB. From the representative P-δ curve, Figure 2a, the CFRP exhibited a typical brittle behaviour with a sudden drop in the critical load point of interlaminar fracture and subsequent smaller load drops. In contrast, the CFRP-PU showed a continuous crack growth with a smoother load slip, giving an indication of plastic deformation at the interface and generally more ductile behaviour. The CFRP-SB and the CFRP-CC specimens followed similar crack propagation behaviour, with more abrupt load drops indicative of more brittle behaviour. The load drops tended to behave in a more stick-slip manner with an immediate arresting of the crack, Figure 2b. This could be also attributed to the existence of the polyester net in the adhesive film, which could act as a local alternating energy store–release structure.

**Figure 3:**
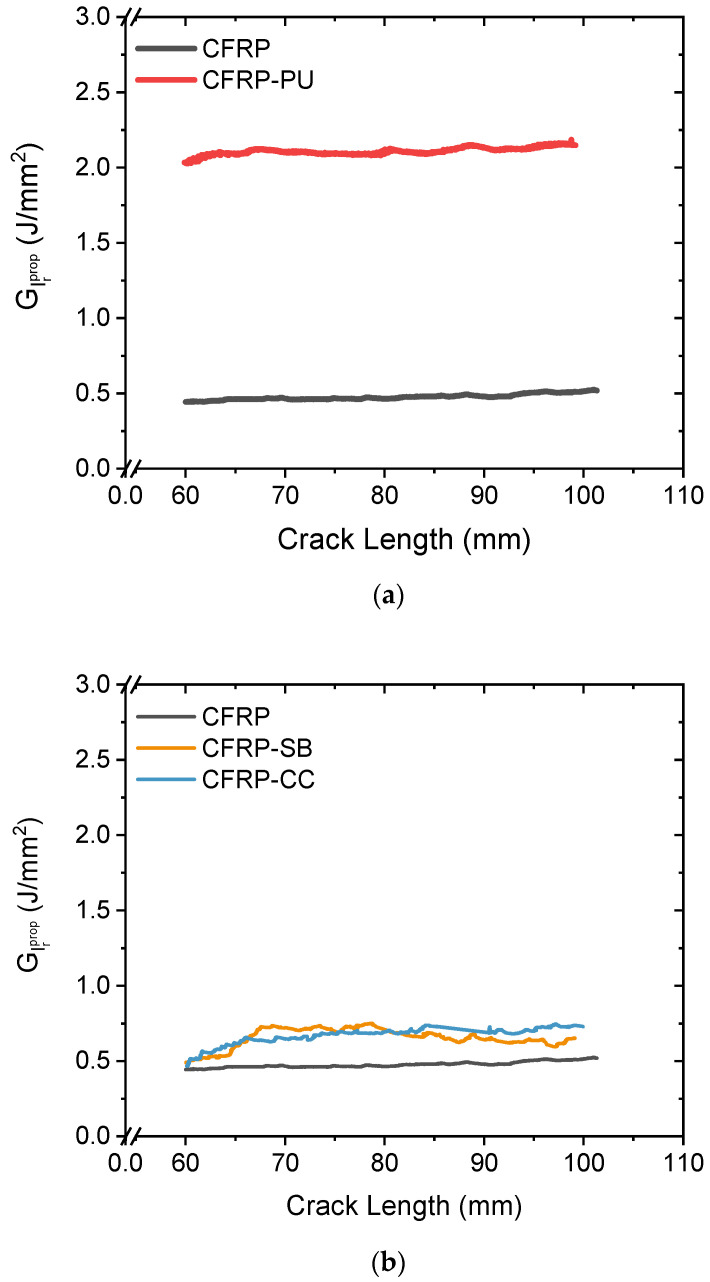
Representative mode I interlaminar fracture toughness in the crack propagation area for the CFRP compared with the (**a**) CFRP-PU; and (**b**) CFRP-SB and CFRP-CC.

The delamination grew from the insert towards the end of the specimen and was highly affected by fibre bridging between the arms of the specimens. The R-curves, Figure 3a,b, show the representative propagated mode I interlaminar fracture toughness values, $G_{I_{r}^{prop}}$, for the comparative cases after the initiation of the crack propagation. For both the CFRP and the CFRP-CC, there was a slight increase in the $G_{I_{r}^{prop}}$ in the crack propagation phase of the test due to the fibre bridging effect, Figure 3b. This contrasted with the behaviour of the CFRP-PU, Figure 3a, and the CFRP-SB, where the fibre bridging effect was significantly lower, and the values for the propagated $G_{I_{r}^{prop}}$ were slightly decreased with respect to that in the crack propagation phase.

**Figure 4:**
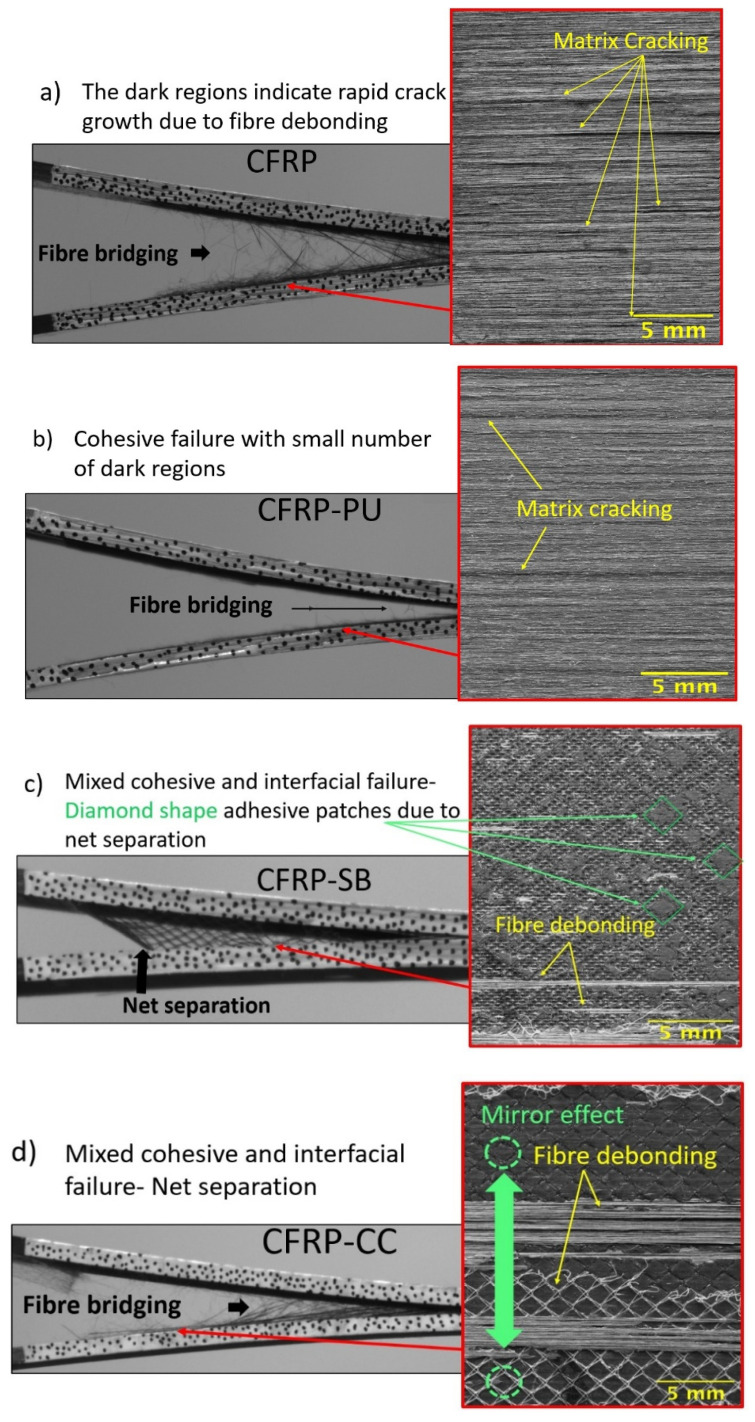
Photographs of failure mechanisms due to peeling forces (DCB) for the (**a**) CFRP, (**b**) CFRP-PU, (**c**) CFRP-SB and (**d**) CFRP-CC.

In general, fibre bridging depends on various parameters, such as weak interfaces and fibre nesting [47]. For instance, as the crack propagates, secondary deformations can occur just below and above the crack plane direction, which leads to localised debonding of the fibres. Figure 4 represents side view captures shots from the recordings of the video gauge extensometer, and images of the fractured surfaces taken with a Nikon D3500 camera. The dark regions of the photograph in Figure 4 indicated rapid crack growth due to matrix cracking. It was seen that the CFRP specimens displayed extensive fibre bridging, Figure 4a, in comparison with the CFRP-PU, Figure 4b, where the fibre bridging was barely noticeable. Smoother behaviour was observed for the CFRP-PU specimens as their thermoplastic nature contributed to a plastic deformation, indicating very good chemical bonding between the two polymeric phases, which featured epoxide and urethane groups, respectively, showing agreement with previous findings [48,49,50]. Finally, cohesive failure for the CFRP-PU specimens was detected, where the PU film remained bonded to both fracture surfaces.

The CFRP-SB and CFRP-CC specimens, Figure 4c,d, behaved in a contrasting fashion. Fibre bridging behaviour was observed with high crack step propagations in both types of specimens, which led to partial loss of detection of the crack propagation in some of the tested CFRP-CC samples. When using the extensometer, it was observed that the sudden crack propagation could sometimes be difficult to detect, especially in brittle fractures. From the P-δ curves shown in Figure 2b, specimens produced by both the manufacturing processes indicated similar behaviour with sudden load slips. For the CFRP-SB, load slips were observed in almost all tested specimens. In Figure 4d, interfacial failure was detected, and in various areas of the CFRP-CC specimens, cohesive failure was also recorded. Due to separation of the polyester net, a mirror effect was observed. CFRP-SB specimens behaved similarly, with less fibre bridging and more areas of cohesive failure, especially as shown in Figure 4c, where one can see diamond-shaped cohesive areas due to polyester net separation during the test.

**Figure 5:**
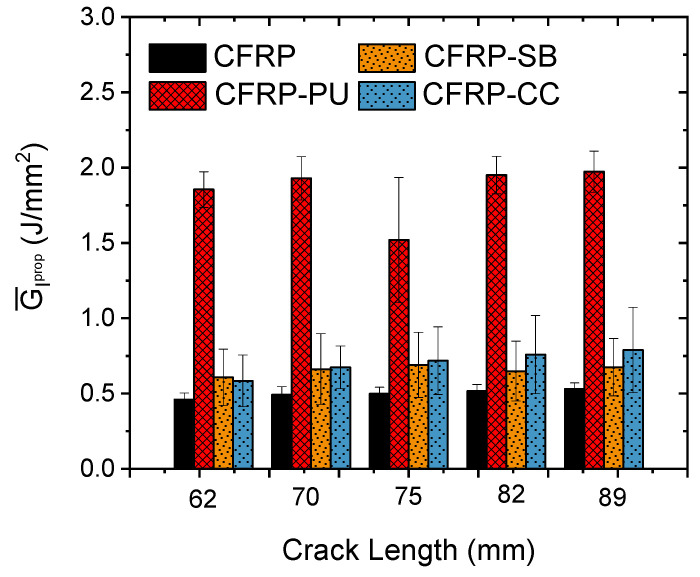
Average mode I interlaminar fracture toughness at different crack lengths for all the cases.

To evaluate all the collected data, an analysis of the average propagated interlaminar mode I fracture toughness, $G_{I_{r}^{prop}}$ for all the specimens was carried out. Figure 5 presents the average $G_{I_{r}^{prop}}$. at different crack propagation lengths after the initiation point. Following the same trend as the representative specimens above, the introduction of a single polyurethane film as well as a single toughened epoxy film measurably improved the interlaminar fracture toughness. The adhesion and the failure mechanisms were affected by the manufacturing process. For example, CFRP-SB tended to show more cohesive failure behaviour than CFRP-CC. A reason for this was because the co-curing process introduced mechanical interlocking between the two epoxies and led to more zones of interfacial failure [49,50]. In the following section, an analysis of the fracture surfaces of specimens from ENF tests is presented to develop a deeper understanding of underlying failure modes.

### 3.2. End Notch Flexure (ENF)

**Figure 6:**
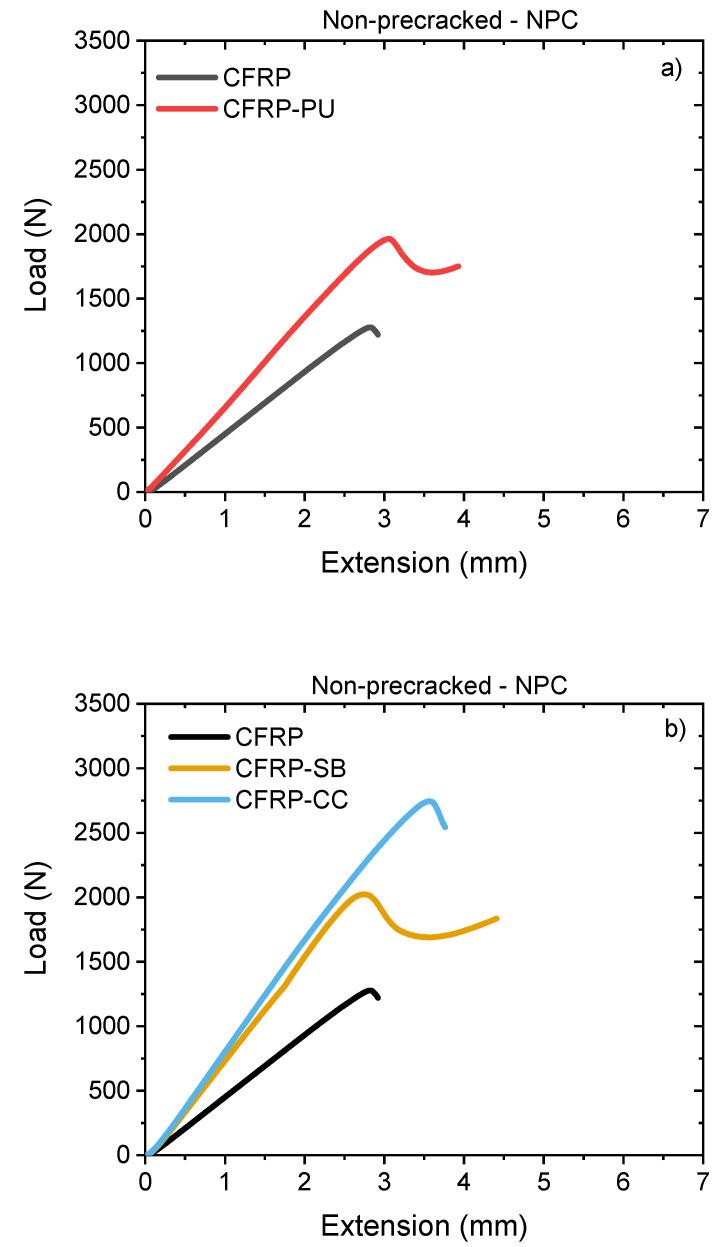

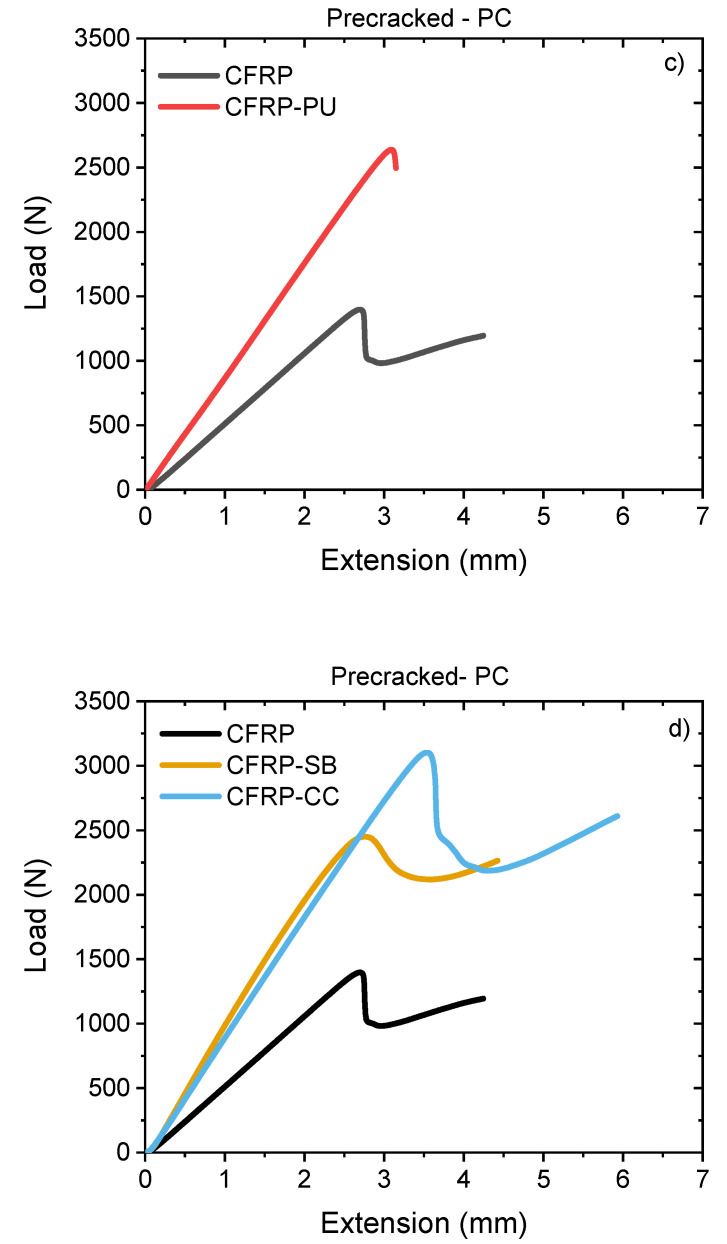
NPC R-curves curves (top) and PC R-curves (below) for (**a**) CFRP compared with CFRP-PU, NPC curve, (**b**) CFRP compared with CFRP-SB and CFRP-CC, NPC curve, (**c**) CFRP compared with CFRP-PU, PC curve and (**d**) CFRP compared with CFRP-SB and CFRP-CC, PC curve.

The mode II interlaminar fracture toughness was measured for all the cases from the load–displacement, P-δ, curves, and is presented in Figure 6. Calibration compliance and data reduction were performed to correct the values according to the test standard. The calculations for the mode II interlaminar fracture toughness in shear were performed with the modified beam theory (MBT). From the P-δ curves below, the reference CFRP specimens had the lowest load capacity, compared to the laminates with the PU film, for both the NPC and PC curves, Figure 6a,c, respectively.

The CFRP-PU, CFRP-SB and CFRP-CC laminates showed higher mode II values than those of neat CFRP laminates, Table 2. More precisely, CFRP-PU had higher mode II toughness than the CFRP, but lower toughness compared to CFRP-CC and only statistically equivalent toughness to CFRP-SB. In both data sets NPC and PC, Figure 6b,d, respectively, CFRP-SB specimens had lower mode II toughness compared with the CFRP-CC, which had the highest mode II toughness than the CFRP. The CFRP-CC showed extremely good resistance to mode II fracture compared to the CFRP-SB, which indicated that the interlocking mechanisms that developed between the two epoxies because of the co-curing process, could be a significant factor in failure due to shear.

**Figure 7:**
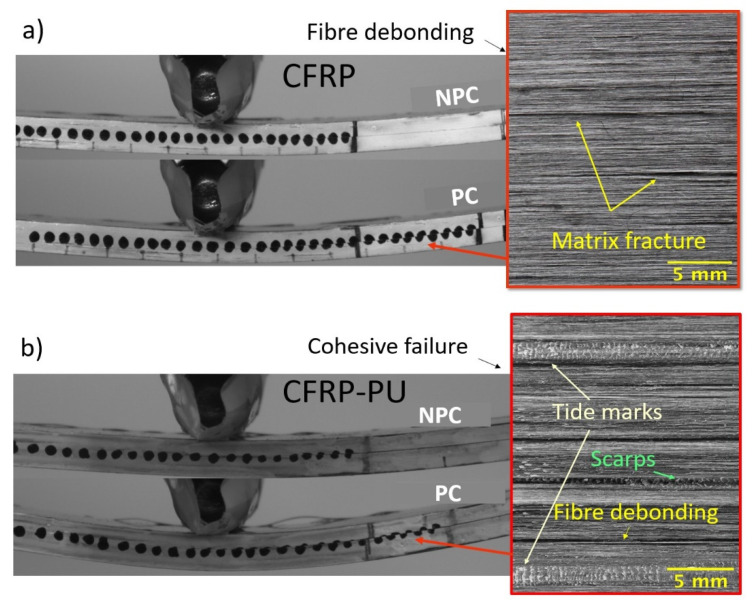

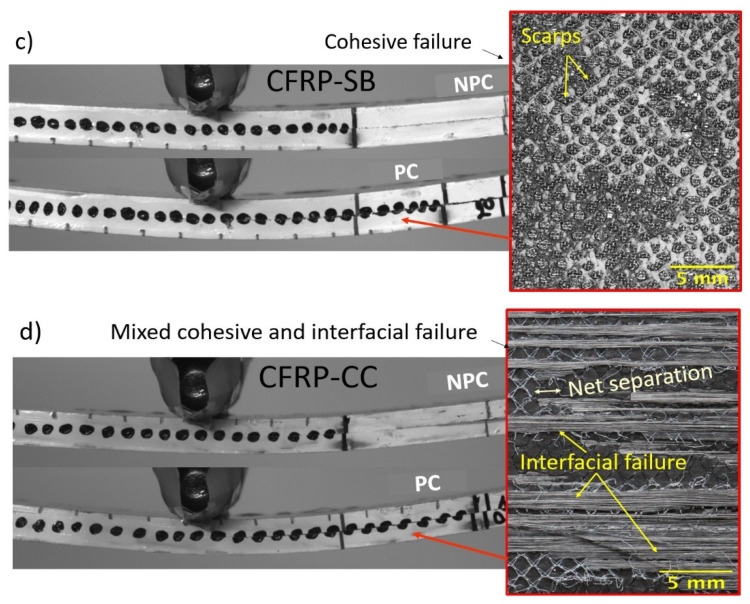
Shear effect due to ENF test for the (**a**) CFRP NPC (top) and PC (bottom), (**b**) CFRP-PU NPC (top) and PC (bottom), (**c**) CFRP-SB NPC (top) and PC (bottom), (**d**) CFRPCC NPC (top) and PC (bottom).

Visually, mode I fracture surfaces were rough in texture because of fibre bridging, while mode II fracture surfaces were smoother, since under shear loading, the fibres fractured either in compression or tension depending on the relative movement of the surface. Figure 7 represents the NPC and PC side view captures shots from the recordings of the video gauge extensometer, and images of the fractured surfaces taken with a Nikon D3500 camera. In general, plastic deformation and higher deflection were observed for CFRP-PU and CFRP-CC, Figure 7b,d, unlike the other two cases, CFRP, Figure 7a, and CFRP-SB, Figure 7c, where their deflection was significantly less. Matrix cracking, dark areas and loose fibres were detected for the CFRP specimens as observed in Figure 7a.

In contrast with the CFRP-PU, Figure 7b, scarps and tide marks were observed on the fracture surfaces of the specimens, which are characteristic of thermoplastic behaviour. Additionally, areas with fibre debonding could be seen due to the opening of the specimen, but overall cohesive failure characterized this type of specimens. Similarly, cohesive failure was observed for the CFRP-SB specimens along with visible areas of scarps due to the net of the adhesive film, Figure 7c. In the case of CFRP-CC, multiple failure mechanisms could be seen. There were areas with cohesive failure, but also areas where the crack propagated, causing interfacial failure. The net separated in a similar manner to that observed in the mode I test. Thus, it is questionable whether the net used for the co-curing manufacturing process is an asset or not, though it could possibly contribute to higher mode II properties.

**Figure 8:**
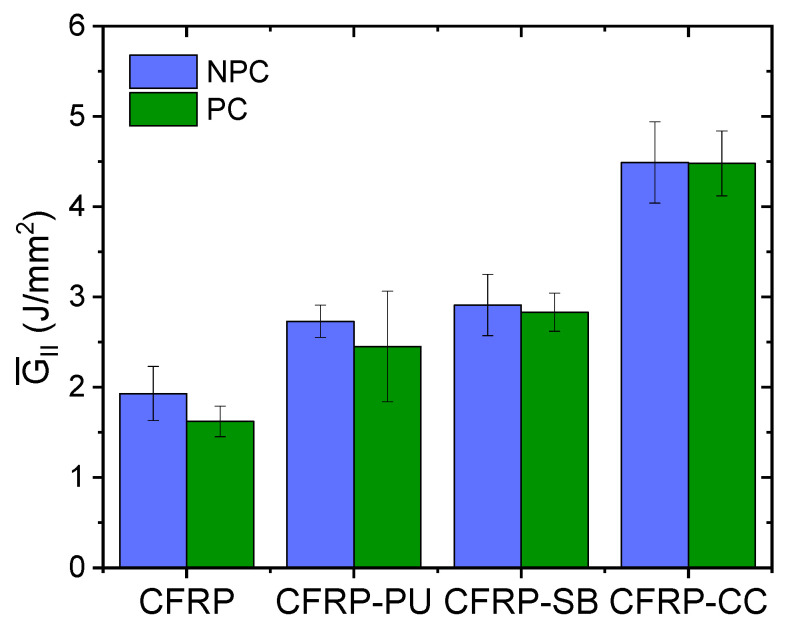
Average mode II interlaminar fracture toughness for all the cases, NPC and PC.

Figure 8 presents the average interlaminar mode II fracture toughness, R-curves, for all the cases for both the NPC and PC curves. The NPC shows slightly higher toughness values than the PC for all the examined laminates. On average, the CFRP recorded the lowest values in mode II while the CFRP-CC values were 177% greater than those of the CFRP. Similarly, CFRP-PU and CFRP-SB resulted in significantly higher average values for both the NPC and PC curves (51% and 75%, respectively) compared to the CFRP.

### 3.3. Scanning Electron Microscopy (SEM)

**Figure 9:**
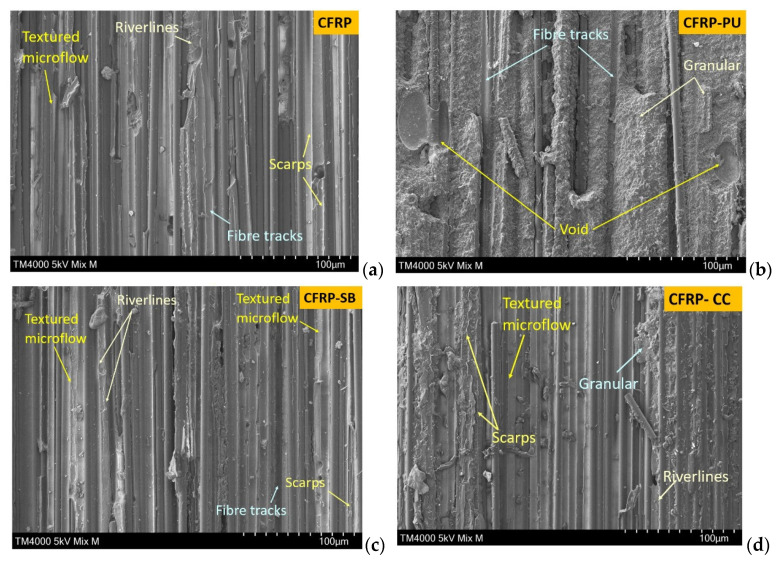
SEM images from DCB fractured surfaces from left to right (**a**) CFRP, (**b**) CFRP-PU, (**c**) CFRP-SB and (**d**) CFRP-CC.

Figure 9a shows the surfaces of failed CFRP mode I, DCB, specimens. Fibre tracks, textured microflow and scraps dominated the surface indicating typical brittle behaviour. Fibre debonding and matrix cleavage were the two dominant fractographic features in this case. Typically, the fractures grew along the fibres, then spread into the matrix as a textured microflow, and then converged with each other. Finally, due to the crack propagation, ribbons and riverlines were detected in CFRP fractured surfaces. In contrast, the microstructure of CFRP-PU exhibited a rougher fracture surface, Figure 9b. The fractographic behaviour of this material tended to be dimpled or granular because of the tough nature of the thermoplastic film. As a result, the fracture surface was characterised by a tortuous crack path with visible fibre tracks, which also provided evidence that the adhesion between the polyurethane, the epoxy resin and the carbon fibres was very good. Scarps, riverlines and textured microflow were observed in both the CFRP-SB and the CFRP-CC specimens, Figure 9c,d, respectively. Although textured microflow was less evident in the latter, the brittle characteristic fractographic behaviour was still observed, especially in the fibre track areas. Lastly, in areas of the CFRP-CC specimens, granular surfaces were observed, indicating that the adhesive had a toughening character.

**Figure 10:**
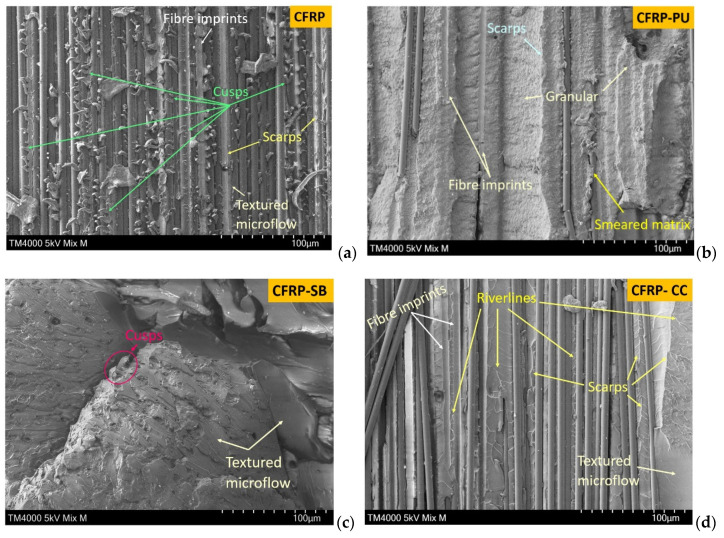
SEM images from ENF fractured surfaces from left to right (**a**) CFRP, (**b**) CFRP-PU, (**c**) CFRP-SB and (**d**) CFRP-CC.

In mode II, ENF specimens, the CFRP fractographic surfaces resulted in cusps (or hackles), flakes and textured microflow, Figure 10a. According to the literature [51,52,53], the cusps form in an S-shape that detach from the surface and thus propagate the shear fracture. The cusps then coalesce adjacent to the plane of the fibre, which results in one surface with cusps and the second surface showing scars. Typically, the cusps develop in the opposite direction of the shear deformation. In the case of the CFRP, the cusps were the dominant phenomenon. Figure 10b presents the fractured surface of the CFRP-PU after ENF testing. In this case, the micro-surface fracture was characterised by a deformed matrix that was smeared across the fractured surfaces. Furthermore, no cusps could be seen as the cohesive failure mechanism of the thermoplastic CFRP-PU did not allow them to develop. Fibre imprints and scarps were formed along the direction of the shear loading. The granular areas could be clearly seen in almost all the surfaces of the CFRP-PU specimens.

In contrast, coalescence due to void formation was observed in the CFRP-SB specimens, Figure 10c. The polyester net that existed in the adhesive film resulted in a pure cohesive failure as observed in the previous section; therefore, the lack of fibre observation is particularly obvious in the SEM images of the CFRP-SB mode II specimens. In this system, cusps were difficult to observe because of the post-curing process. Scarps and textured microflow, however, could be seen. Plastic deformation or viscoelastic deformation in the polyester net could lead to cohesive failure in the adhesive zone. Riverlines, scarps and textured microflow were exhibited in the CFRP-CC microstructure, Figure 10d, indicating a highly deformed matrix. There were also some imprints of fibres. In this case, there was clear evidence of crack growth that followed the riverlines. The parameters that influenced the fracture surfaces were many, with the most important being the loading and unloading rate in the case of ENF (ASTM D7905).

## 4. Discussion

From the experimental study, significant increases in mode II toughness were observed for all the optimised laminates CFRP-PU, CFRP-SB and CFRP-CC, while only CFRP-PU showed a significant increase in mode I toughness. The results for the optimised laminates are compared to those of the reference, CFRP. G_Ic_ is a geometry dependent property, which is influenced by the thickness of the adhesive layer, while G_II_ is an estimated value based on the material parameter [54]. This study focused on the adhesive properties of the examined laminates and their interfacial response to both shear and peel stresses rather than the influence of the thickness on the laminates’ properties. Relative to the overall thickness of specimens, similar film thicknesses were used, but in this study, we examined single use of films as adherents and their adhesive properties.

### 4.1. Mode I Performance

It was clear that CFRP-PU adhered very well to both the fibres and the matrix; therefore, this has resulted in significantly higher values for mode I toughness and lower fibre bridging compared with CFRP-CC and CFRP-SB. The very high increase in mode I toughness delivered by non-netted CFRP-PU. Equivalent mode I toughness, shown by netted CFRP-CC and CFRP-SB, could be explained by the fact that the presence of netting was not an advantage in mode I because of the lower relative contribution of frictional resistance during peeling compared with adhesion strength, since normal rather than tangential forces predominated. Indeed, the presence of a net may have been a disadvantage, as it could feature more incipient adhesive failure sites. This was likely to explain the high increase in mode I toughness of CFRP-PU, contrasted with no significant increase in CFRP-CC and CFRP-SB mode I toughness. This means that a continuous adhesive film, such as PU without a net, could perform better in mode I than a film with an embedded net, if the continuous film had high adhesive strength. Additionally, thermoplastics such as PU tend to distribute the energy locally, also reported elsewhere [55,56], while thermosets tend to distribute the energy rapidly to the whole specimen.

### 4.2. Mode II Performance

For the three enhanced CFRP laminates (-PU, -SB and -CC), the interpenetration network—created by chemical interlocking of functional groups during curing—was critical to effective bonding of the film with the matrix. However, mode II sliding resistance was governed not only by the adhesion of the film, but also by the resistance of a much larger surface area of the material compared to mode I, where there was a high stress concentration over a smaller front due to peeling. This explained why there was a much higher increase in mode II toughness for CFRP-CC and CFRP-SB compared with CFRP, due to the contribution of net sliding friction, which was not present in CFRP-PU. Therefore, despite the excellent adhesion of CFRP-PU, it delivered a lower improvement in mode II toughness compared with the laminates with netted film. This is supported by Da Silva et al. [57], who reported an increase in shear strain due to the addition of a net compared to a non-netted control bismaleimide adhesive. This supports our hypothesis that there was better enhancement of shear strength for net-containing adhesive films compared to the non-net containing PU film. Thus, for mode II-driven applications, the net becomes an overwhelming asset because of its contribution to enhanced shear resistance as the interfaces shear over each other, since the increased net roughness increases the required energy of shear.

### 4.3. Comparison of Co-Curing and Secondary Bonding of the Epoxy Adhesive Film

There are many parameters that affect the performance of bonded composites, i.e., adhesive layer thickness, netting, type of bonding process, etc. [58]. A co-cured interface will normally be stronger than an interface where a pre-cured surface is bonded to a fresh adhesive. During co-curing, the adherend cures together with the matrix under the same conditions; thus, there is more covalent bonding compared to SB where a pre-cured surface offers fewer functional groups for chemical bonding with the adhesive [38,59]. This explains the higher increase in mode II toughness for CFRP-CC compared with that for CFRP-SB. Secondly, it is known that post-curing can degrade the adhesion properties at an interface: this is due to the fact that since the laminate epoxy is already cured, further heat treatment can possibly result in degrading its crosslinking state both within itself and with the fresh adhesive. This is consistent with findings of previous works [60,61], which have proven that, depending on the post-cure temperature, post-curing gradually decreases the mechanical properties of adhesive bonded composites, i.e., it can result in lower interlaminar shear strength. However, in the present case, a processing temperature of 120 °C is unlikely to have promoted a significant degree of chemical bond degradation.

### 4.4. Hold and Release Mechanism of the Epoxy Film Due to the Net

**Figure 11:**
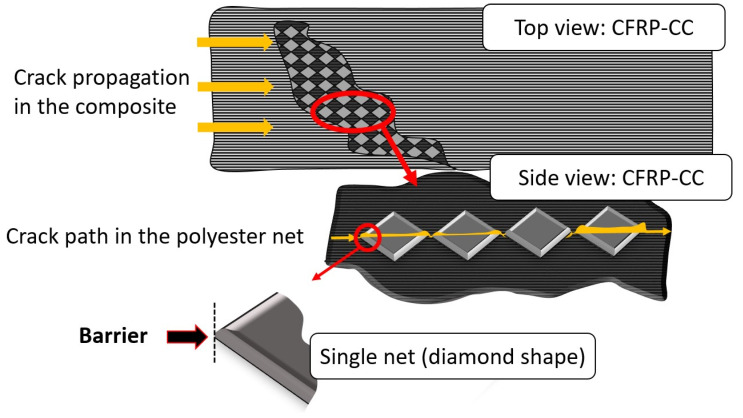
Illustration of the crack propagation of mode II in CFRP-CC.

In Figure 11, an illustration of the CFRP-CC behaviour in mode II is presented. The crack propagation due to shear loading meets a ‘barrier’ at the polyester net. A local energy ‘hold and release’ mechanism takes place, (see stick-slip behaviour in Figure 2b) due to the structure of the net in the loading direction. Similar behaviour was observed by Parvatareddy et al. [62], where the scrim cloth of the adhesive used played an important role in energy dissipation by providing weakened sites.

The behaviour in Figure 11 demonstrates a need for deeper micromechanical study of such adhesive films with embedded nets to better understand how they affect the interlaminar behaviour of bonded composite laminates.

## 5. Conclusions

In this study, the adhesive and interlaminar fracture toughness properties were measured for hybrid carbon fibre reinforced polymers (CFRP) with toughened interleaves. Two different single films were introduced in the CFRP to increase the interlaminar fracture toughness: a thermoplastic polyurethane film, and an epoxy adhesive film VTFA400 featuring a polyester net. The latter was inserted in the CFRP using two different manufacturing processes: a typical co-cured layup process and a secondary bonding process. On average, the adhesion between the CFRP epoxy laminate and the PU film, CFRP-PU, led to significant increases of 294% in mode I toughness and of 51% in mode II toughness. By contrast, the laminates with epoxy adhesive films, CFRP-SB and CFRP-CC, respectively, showed mode I toughness values that were equivalent to that of CFRP on average. Furthermore, the manufacturing process and the polyester net of the adhesive film had a significant effect on the interlaminar properties obtained. The average Mode II toughness of the CFRP-CC increased by 177% over baseline, which was far greater than the equivalent increase for CFRP-SB of ca. +75%, because of the superior adhesive bonding between the two epoxies during co-curing. In addition, the influence of the polyester net could also be a factor in the toughening behaviour of both the above epoxy-toughened laminates, as its design could lead to localized alternating store-release energy behaviour. Initial evidence for this behaviour was observed in the fractured surfaces, but further study needs to be carried out to verify this. The net also drove higher increases in mode II toughness for the CFRP-CC and CFRP-SB compared with CFRP-PU because of its contribution to increased sliding friction. In contrast, CFRP-PU delivered a high increase in mode I toughness since the contribution of adhesive strength was more significant over a smaller surface area of peeling relative to mode II sliding. Secondly, the frictional contribution of the net during sliding was not as prominent in the mode I peeling mode. The laminate fracture surfaces were analysed using SEM images. The failure behaviour was characterised in each case by different morphologies with respect to both type of insert and the manufacturing route. Good adhesion and high fracture toughness are preferred for a variety of applications especially for bonded structures and where impact energy absorption is a prime requirement.

## Figures and Tables

**Table 1:** Thickness, density, and FVF for all the manufactured laminates.

Cases	Thickness (mm)	Density (g/cm^3^)	Fibre Volume Fraction (%)
CFRP	4.02 ± 0.11	1.49 ± 0.01	51
CFRP-PU	4.03 ± 0.04	1.49 ± 0.02	50
CFRP-SB	5.00 ± 0.25	1.48 ± 0.02	51
CFRP-CC	4.66 ± 0.10	1.51 ± 0.01	54

**Table 2:** Critical interlaminar fracture toughness properties of all the tested cases (N/A = no statistical difference).

Cases	$\mathrm{Mode}\;I,\;{\bar{G}}_{Ic}\;(J/{\mathrm{mm}}^{2})$	$\mathrm{Mode}\;\mathrm{II},\;{\bar{G}}_{IIc}\;(J/{\mathrm{mm}}^{2})$
CFRP	0.36 ± 0.08	1.62 ± 0.17
CFRP-PU	1.42 ± 0.15	2.45 ± 0.62
CFRP-SB	0.43 ± 0.11	2.83 ± 0.21
CFRP-CC	0.37 ± 0.07	4.48 ± 0.36

## Data Availability

The data presented in this study are available on request from the corresponding author.
